# Local design and manufacturing of patient-specific implant using Anatomage Medical Design Studio software: proof of concept - Botswana’s 1st case report

**DOI:** 10.1186/s41205-023-00170-2

**Published:** 2023-03-23

**Authors:** Shathani Nkhwa, Thapelo Montshiwa, Deon de Beer, Gerrie Booysen, Cules van den Heever, Johan Els, Andre Heydenrych, Maikutlo Kebaetse

**Affiliations:** 1 Faculty of Medicine, Department of Biomedical Sciences, University of Botswana, Corner of Notwane and Mobuto Road, Pvt Bag, 00713 Gaborone, Botswana; 2 Sidilega Private Hospital, Orthopaedic Surgery, P.O. Box 70905, Gaborone, Botswana; 3 Free State, Centre for Rapid Prototyping and Manufacturing, Central University of Technology, Private Bag X20539, Bloemfontein, 9300 South Africa

**Keywords:** Patient-specific implant, 3D printing, Medical design studio, Ollier’s disease, Achilles tendon lengthening, Post-operative knee rehabilitation

## Abstract

**Background:**

Botswana, like most sub-Sahara African nations, uses conventional orthopaedic implants that are sourced from major manufactures in the West. The implants are mass-produced and designed with universal configurations to fit an average patient. During surgery, surgeons thus sometimes bend the implants to match the individual bone anatomy, especially for paediatric patients and those with unique deformities, thus risking implant failure. The purpose of this project was to show the feasibility of developing safe and effective patient-specific orthopaedic implants in a low-resourced market.

**Methods:**

CT Scan slice files of a paediatric patient with Ollier’s disease were used to reconstruct the lower limb anatomy. The resultant files were 3D printed into prototypes that showed severe right knee valgus deformity. The surgeon used the prototype to plan for corrective femoral osteotomy and the required implant. The implant design and planned surgery were subsequently simulated on the Medical Design Studio software for proper fitting before final implant printing. Surgery was then performed, followed by 12 weeks of physiotherapy.

**Results:**

Post-surgical x-rays demonstrated good implant positioning and knee joint alignment. At 18 months of post-surgical follow-up, the child was pain-free, could perform full squats, and ambulation was near-normal, without the use of an assistive device.

**Conclusions:**

It is feasible to develop effective, patient-specific implants for selected orthopaedic cases in a low-resourced country. This work could improve surgical and rehabilitation outcomes for selected paediatric patients and those with severe bone deformities.

## Introduction

Trauma, tumours, congenital abnormalities, and other pathological conditions may lead to bone tissue defects, which may result in significant functional and aesthetic deficits in affected individuals [1]. Metallic surgical implants have been extensively used in the reconstruction, stabilization, and management of orthopaedic injuries [2]. Currently, Botswana, like most developing countries, outsources conventional medical devices such as implants from major manufactures via regional distributors. These include companies that sell medical devices globally, such as Zimmer Biomet and Johnson and Johnson DePuy Synthes, both of which are based in the United States. These implants are mass-produced and designed with universal configurations to fit an average patient. Because these medical devices are not patient-specific, they may not fit some patients, such as the pediatric population [3] and those with severe congenital or traumatic deformity. Computer aided design and medical 3D Printing are well suited for these patients, including anatomic models and anatomic guides. Not only can customized design and 3D printing be performed outside of traditional industry, but also it can be shown to have high utility and have a cost-benefit to the medical organization [4–6]. This balance of cost and benefit is paramount in Botswana and other similar countries – for Botswana, patients who require complex surgery, such as in this Case Report, are often referred to South Africa at a high financial cost to the government.”

Surgeons sometimes improvise for implant mismatch by manually bending implants, such as fixation plates, during surgery to match the individual bone anatomy [7]. A major concern, however, is that manipulation of implants could result in structural/mechanical and functional damage to the implanted device, which could lead toits fatigue-failure, corrosion, and other complications [8]. The bending of implants during surgery has also been reported to be a physically demanding and time consuming process [8].

In Botswana, where deformity assessment and subsequent surgical planning are traditionally based on pre-operative radiographic studies, one concern is that this technique could limit the surgeon’s ability to accurately plan and effectively perform surgical correction, especially in complex cases. The traditionally used approach thus has the potential to increase the risk of anatomical mismatch and subsequently the likelihood of not-so-desirable functional and cosmetic outcomes. We suggest that customised pre-surgical planning could help address these challenges.

To help solve the challenges encountered when using generic implants and traditional approaches to surgery, we explored the use of Medical Design (MD) Studio (Version 1.0, Anatomage, San Jose, CA) along with three-dimensional (3D) printing technology. With advances in medical imaging and computing programming, virtual models of the patient’s own anatomy can be generated from computed tomography (CT) or medical resonance imaging (MRI) scans for evaluation and planning of patient-specific orthopedic procedures [9, 10]. Software, such as Anatomage Medical Design (MD) Studio, can be used for anatomical defect reconstructions and enhanced evaluation and surgical planning. Computer-aided designs (CAD) of the surgeon’s desired implant can then be developed from the reconstruction, based on the patient’s anatomy. Additive Manufacturing technology, better known as 3D Printing, is a manufacturing technology that allows for quick fabrication of either plastic or metallic components with any complex shape, which is enabled by the inherent layer by layer process of material deposition during printing [11–13]. The desired implant designs and proposed surgical procedure can then be simulated before surgery and printing of the implants to ensure perfect patient-implant fit as per the surgeon’s requirement [14].

While a number of studies report work on the use of 3D-printed patient specific implants [12, 15], such literature is very limited in sub-Saharan Africa, with a few published reports from Central University of Technology in South Africa [16–18]. To the best of our knowledge, no clinical use of the Anatomage MD Studio has been reported for pre-surgical planning, design fit assessment and simulations in the manufacturing of patient-specific implants in Botswana and the rest of the continent of Africa.

Through collaboration across biomedical and biomaterials engineering, orthopaedic surgery, and rehabilitation science, we undertook a proof-of-concept project, whose purpose was to develop a safe, effective, and affordable patient-specific 3D printed paediatric orthopaedic implant. The following case report thus describes a novel application in developing a 3D printed patient-specific implant using the MD studio in pre-surgical planning.

## Case presentation

### Patient demographic and history of presenting illness

**Fig. 1:**
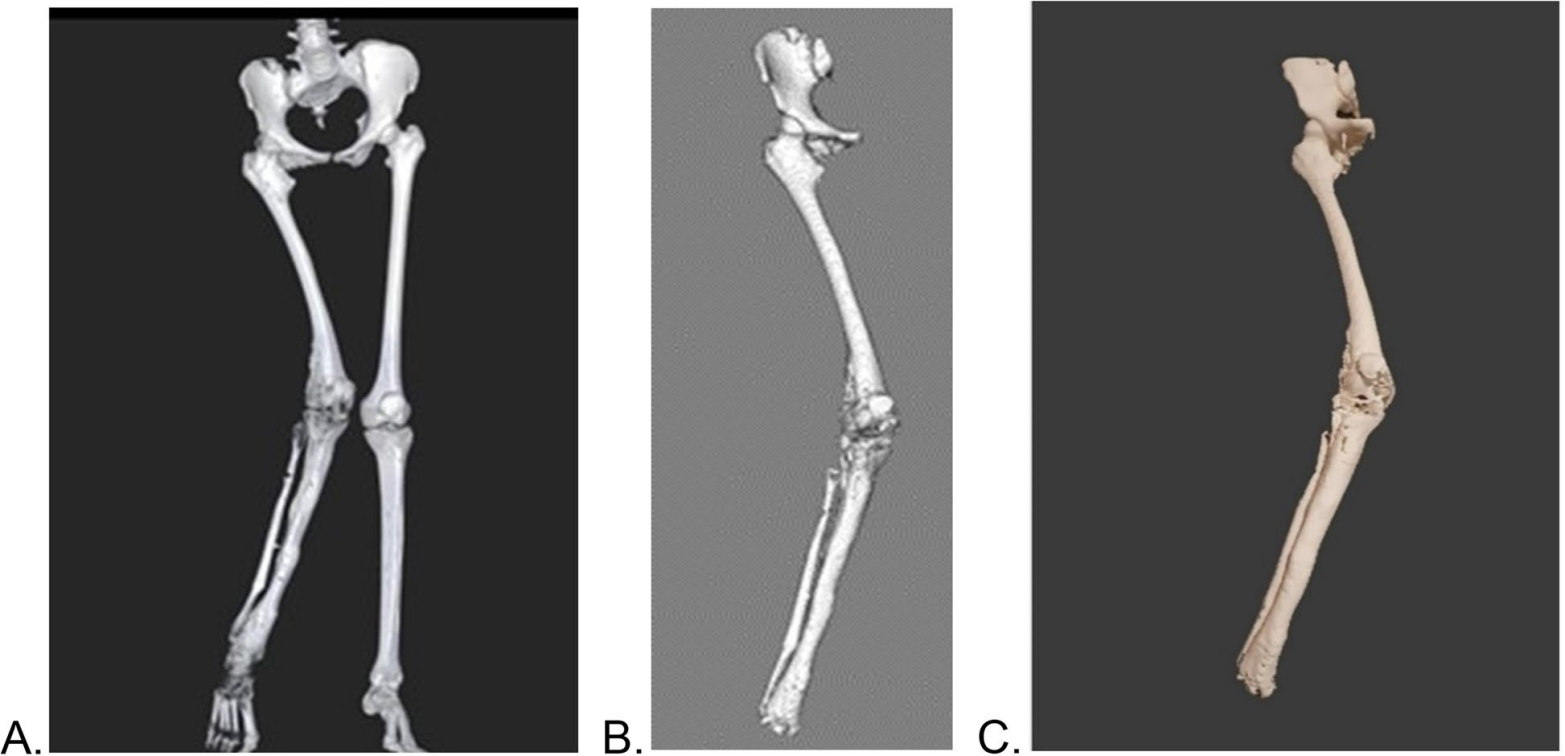
**A**) Pre-surgery CT scan of patient, **B**) Anatomage MD Studio anatomical models of the defect area, and **C**) 3D printed prototype model of the anatomical defect

One (1) female paediatric-adolescent patient was identified by an independent qualified surgeon from an orthopaedic department based at Princess Marina Hospital (the main government referral hospital in Botswana) and a CT scan was obtained (Fig. 1A). The patient was a 14-year-oldchild with Ollier’s disease affecting the right side of her body and resulting in shorter long bones on her right side. The most affected parts were the right femur, tibia, humerus, and forearm: each right limb was ~ 15 cm shorter than the corresponding left limb; there was severe right genu-valgus deformity, and she had moderate right ankle equinus deformity and Achilles tendon shortening. Our focus, however, was the right lower limb because her chief complaint was gait dysfunction that was caused by her markedly shorter leg and valgus deformity.

**Fig. 2:**
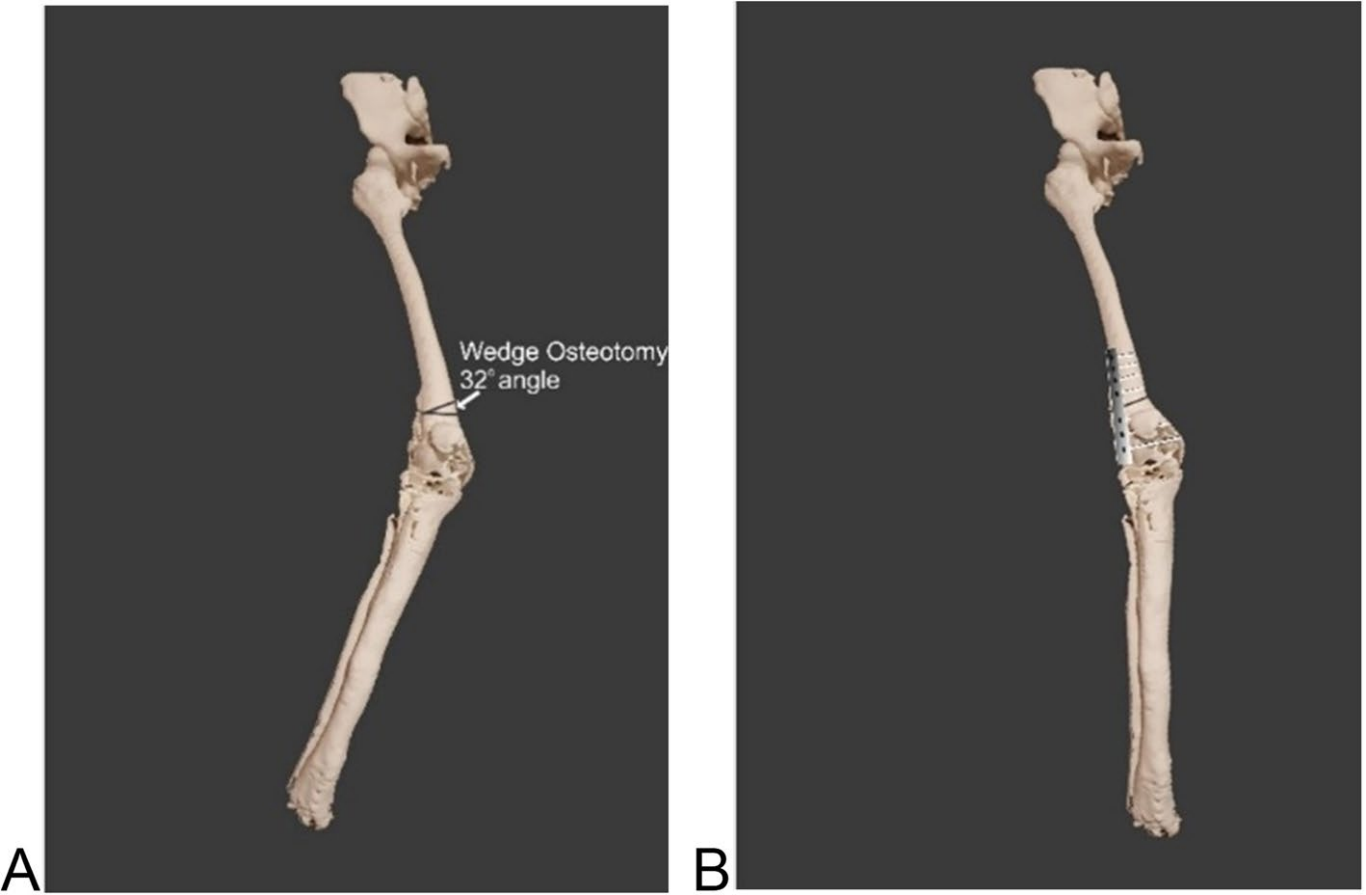
**A**) Surgical plan by surgeon using Coreldraw, **B**) Desired implant design concept by surgeon

The child had initially been offered a shoe-raise, which she found too big and heavy and thus cumbersome, cosmetically unattractive, and not adequately improving her gait. She subsequently underwent surgical limb lengthening using the Ilizarov distractive osteogenesis technique, which reduced her limb length deficit by about 7 cm. The surgeon was, however, unable to correct the genu valgus deformity (~ 20-deg lateral deviation of the tibial shaft from the femoral shaft in anterior view) at the same surgical setting. This deformity remained quite significant and problematic for her, both functionally and cosmetically. As treatment for that deformity, she was offered corrective osteotomy and fixation with “custom-made” plate and screws.

### Pre-operative clinical and radiology findings

The patient was assessed pre-operatively both by the surgeon and rehabilitation scientist. The main findings of interest were: the child weighed 41.3 kg and was 143 cm tall; she was independent in personal care, learning activities at school, all transfers (getting up from sitting on the floor, chair, and in and out of bed), walking for up to 1 km using elbow crutches, and stair climbing using railing/crutches; prolonged standing activities and walking distances greater than 200 m were said to cause exhaustion, low back pain (8/10), and sometimes falls; during walking, her trunk moderately swayed to the right side and she compensated with leftward lateral trunk flexion; in supine position, there was a 20-degree angle between the long axes of the right femoral and tibial shafts, representing valgus angle and deformity; there was functional knee flexibility, moderate right ankle equinus deformity, 0-degree dorsiflexion when sitting with knees and hips flexed to 90 degrees, and the right lower limb muscles were slightly weaker at 4/5; and overall, she scored 62/80 (77.5%) on the lower extremity functional scale (LEFS) [19].

## Materials and methods

### Pre-operative digital and physical planning workflow

**Fig. 3:**
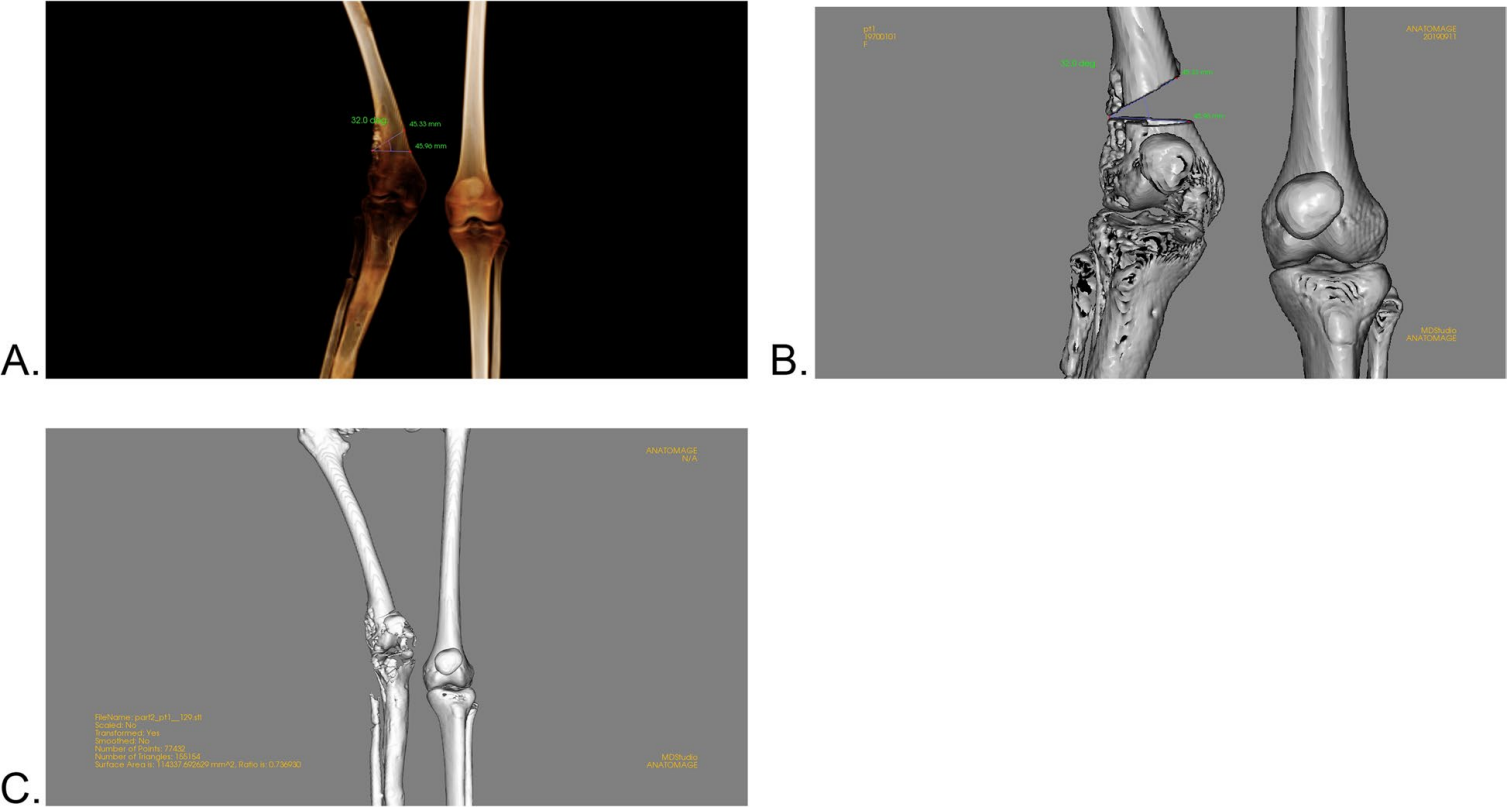
Images **ABC** show simulation of surgical plan on Anatomage MD studio

The workflow started with the conversion of slice files to Digital Imaging and Communications in Medicine (DICOM) format, obtained from the lower body via conventional CT scan with slice thickness of 1 mm (40 mAs, 135 kV). The slice files were converted to DICOM using a DICOM converter (DICOM converter. Ink), to allow loading of slices on MD Studio software. The conversion was necessary because the slice files came in an unreadable file format and not a .dcm format. Using MD Studio, a segmented 3D virtual model of the patient’s lower body anatomy with both soft tissue and bony structures was obtained. A 3D reconstruction model of the patient’s skeletal structure was created (Fig. 1B), exposing the defect area, whose file was saved as a standard tessellation language (STL) file. A 1:1 scale prototype from the STL file was outsourced to Botswana Institute of Technology, Research, and Innovation (BITRI) for 3-D printing (Fig. 1C). The prototype was 3D printed with an EOS Formiga P110 SLS 3D Printer (Krailing, Germany) using PA 2200 powder material. Once the print was completed, excess powder material was removed using compressed air. The surgeon then used the prototype to physically plan the surgery, showing the marks and cuts he would make (Fig. 2A). He then made the cut on the prototype and showed how the correction would appear using Corel draw, hence providing guidance for the implant design (Fig. 2B). The cut and alignment were then virtually simulated on MD Studio to confirm the surgeon’s proposed procedure and outcome Fig. 3. With this confirmation the final design and manufacturing of the implant were outsourced to the Central University of Technology (CUT)‘s Centre for Rapid Prototyping and Manufacturing (CRPM), South Africa.

### Computer-aided fit validation and manufacturing

**Fig. 4:**
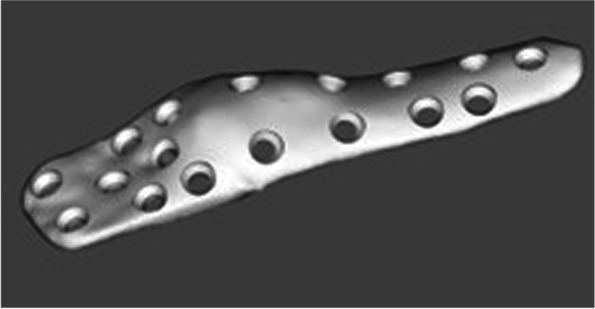
3D models of implant

**Fig. 5:**
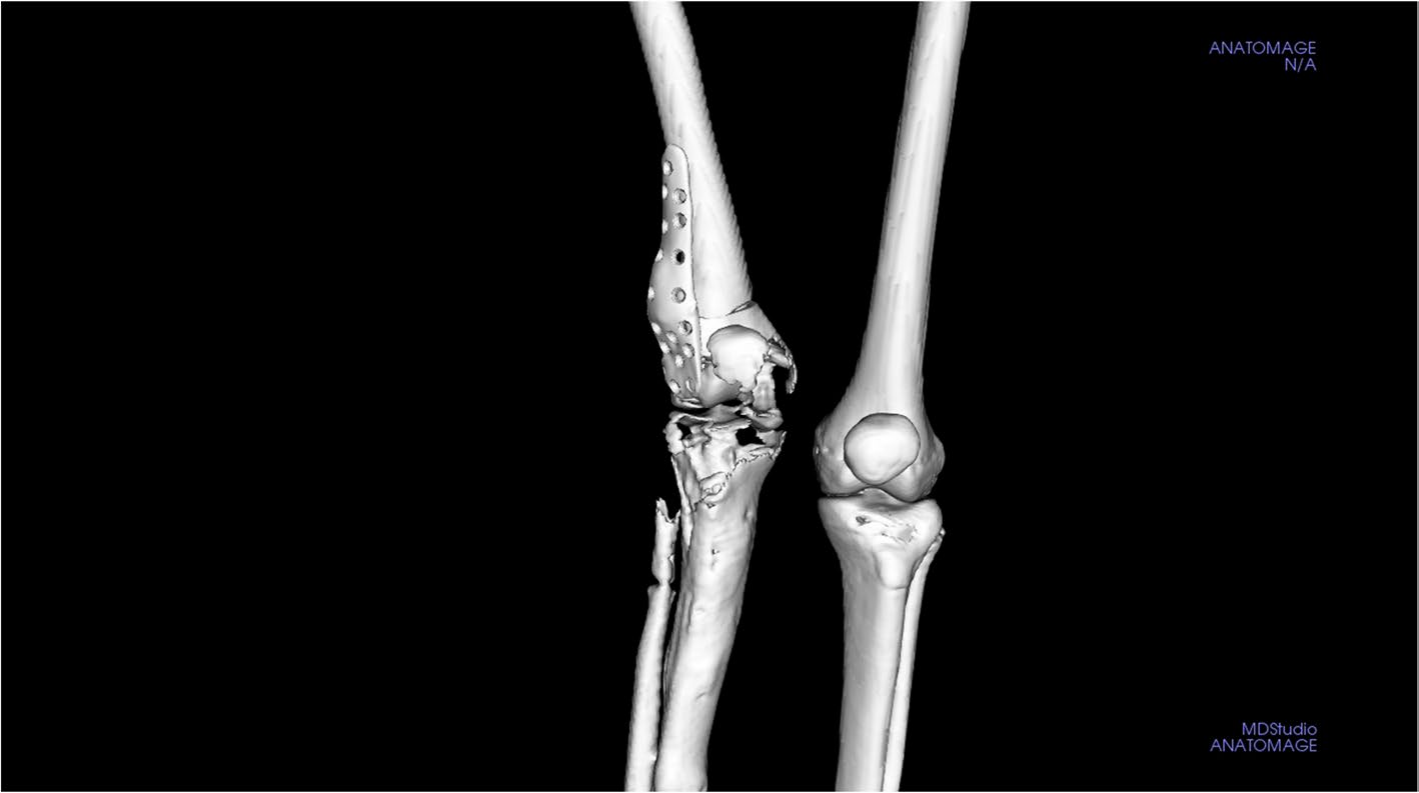
Simulation of implant fit to deformity correction site on Anatomage MD studio

The designed implant file (Fig. 4) was sent to Botswana for fit validation and for approval before manufacturing (Fig. 5). The validation was virtually conducted on MD Studio to assess patient implant fit as per the surgeon’s requirements. Once the surgeon was satisfied, CUT was given a go ahead for the printing/manufacturing of the implant. The implant was printed in titanium (Grade23 Titanium-6-Al-4-V) using CUT’s EOS M290 Metal additive manufacturing facilities. The implant was taken through non-destructive testing CT scanning, heat treatment, polishing, and cleaning before being sent to Botswana. Additional test samples were printed for mechanical destructive testing.

**Fig. 6:**
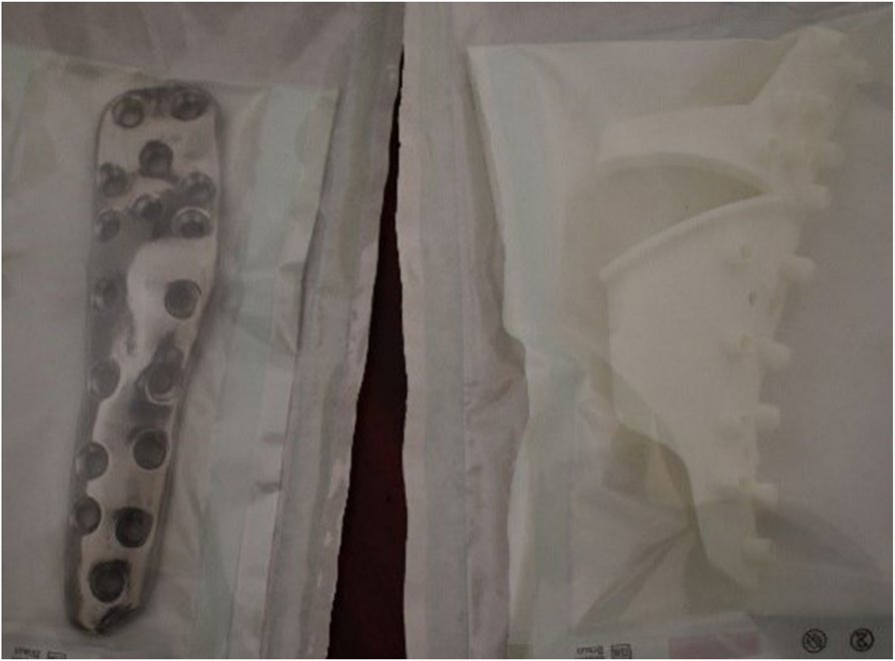
Implant (Grade23 Titanium-6-Al-4-V) and drill guide received from CUT

**Fig. 7:**
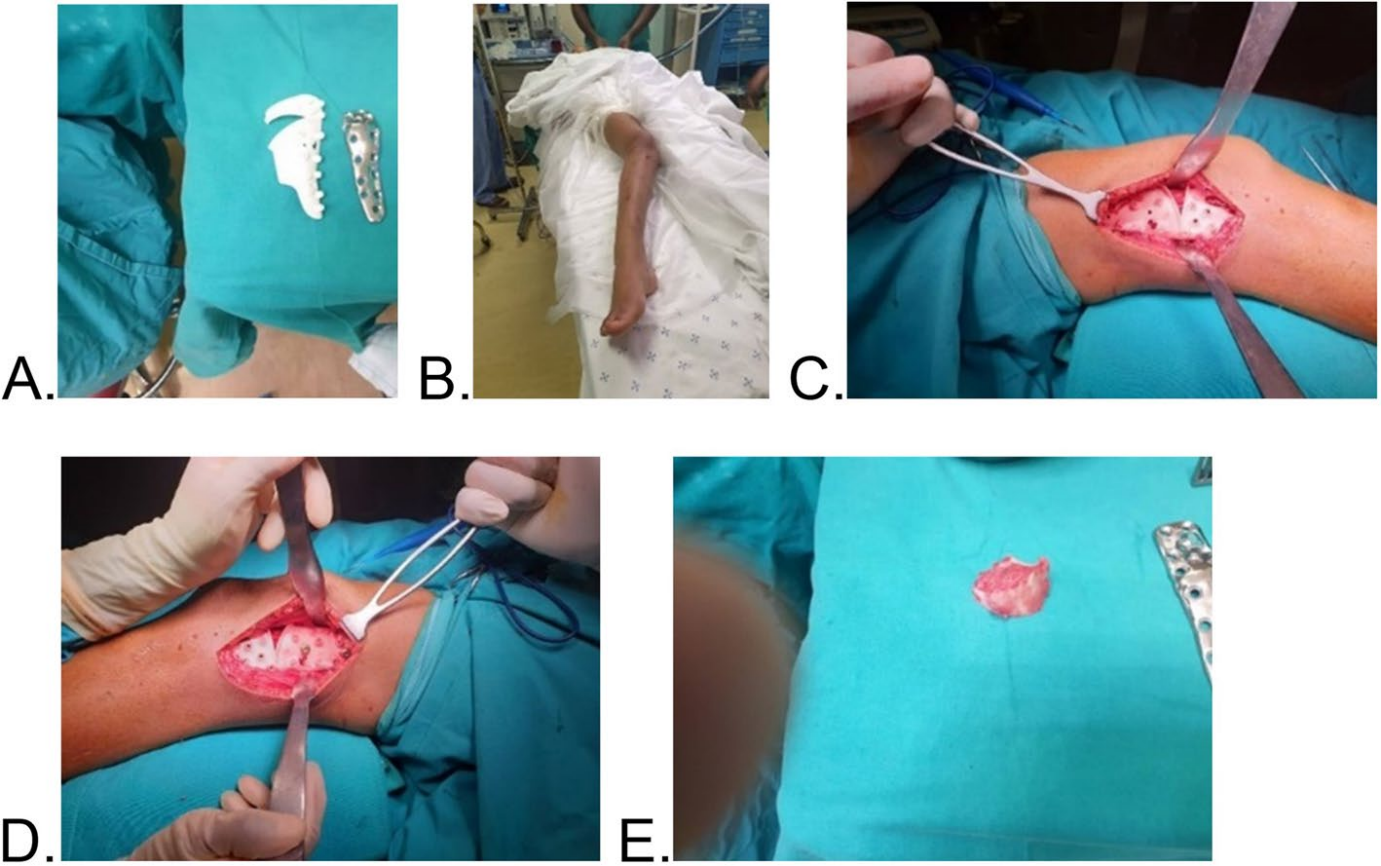
Images **A**-**E** illustrating surgical procedure using developed drill guide and implant

**Fig. 8:**
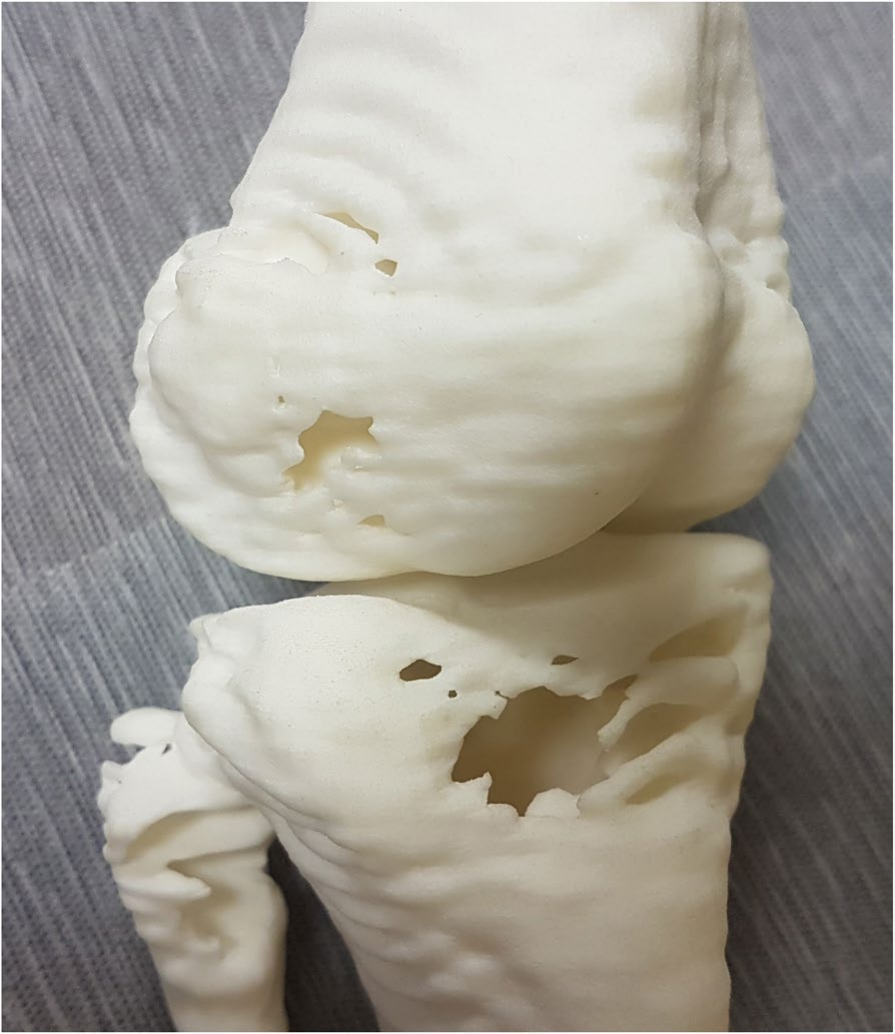
Image of the 3D-printed prototype illustrating ripple-like effects and hollow areas

**Fig. 9:**
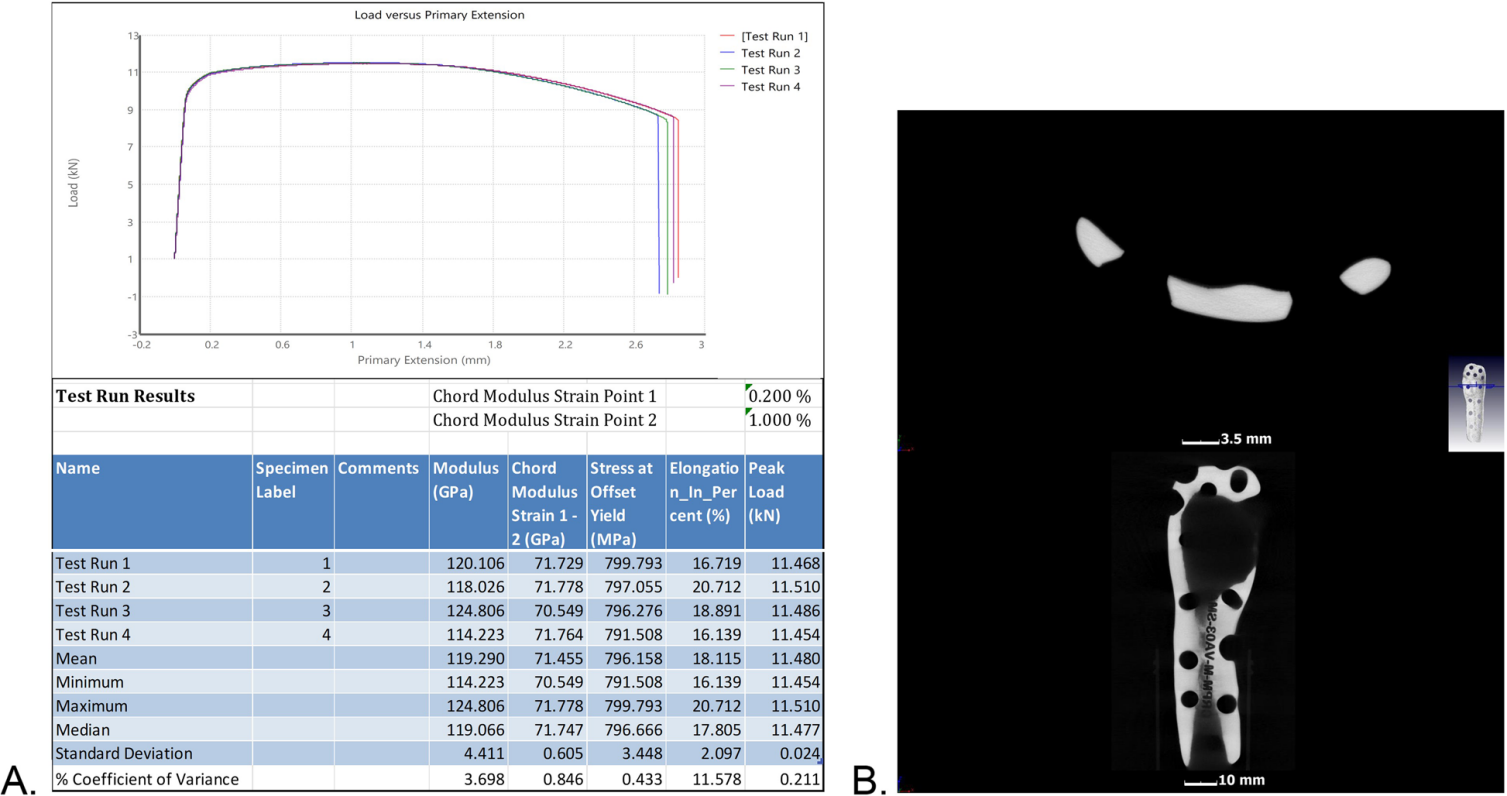
**A**) graph and table presentation of mechanical properties conducted on the destructive test samples, **B**) CT scan of implant showing no microcracks after post processing

**Fig. 10:**
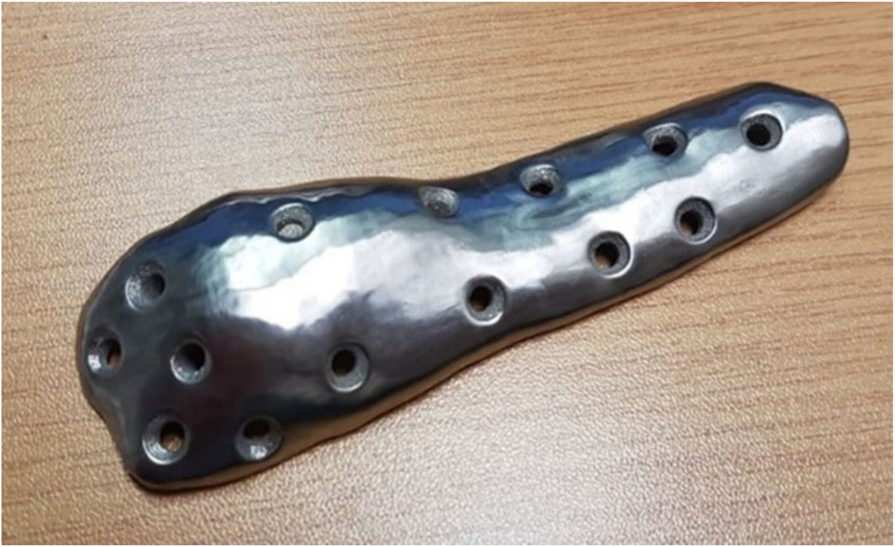
Image of final implant after post processing

**Fig. 11:**
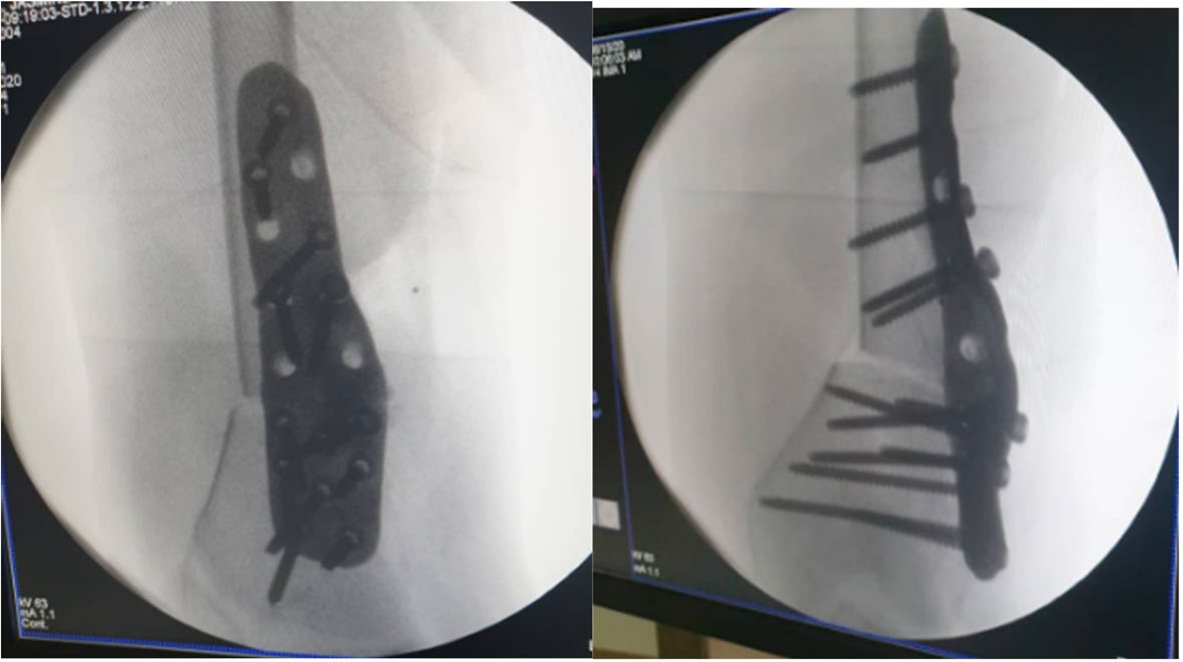
X-ray image of implant positioning right after surgery showing excellent fit

### Surgery

Upon receipt, the implant was visually inspected for any gross defects, and none were found (Fig. 6). The implant and the osteotomy/drill-guide were sterilized via standard hospital autoclave protocols in preparation for the surgical procedure. Surgery was performed under spinal anaesthesia administered by an anaesthesiologist. Sterile aseptic operating technique and a lateral approach to the distal femur were used (Fig. 7). The distal femur was noted to have irregular surface, with cartilaginous-looking masses on the surface, consistent with characteristic appearance of Ollier’s disease enchondromas. 3D-printed drill-osteotomy guide was applied to the distal femur laterally, fitting well to the irregular bone as on the pre-surgical model simulations and trials. The drill-osteotomy guide was temporarily fixed to bone with two 1.8-mm k-wires. Pre-drilling of the screw holes was performed with a 3.2-mm drill bit. Osteotomy was done using an oscillating saw through the pre-determined angle of 32 degrees on the drill-osteotomy guide to create a closing wedge osteotomy. The k-wires and guide were removed, and the closing wedge osteotomy was reduced to achieve alignment. The titanium femur plate was placed and fixated with 4.5-mm diameter titanium screws of corresponding diameters from Depuy Synthesis.

Irrigation of the surgical wound site was done with saline and surgical wound closure archived in anatomical layers using vicryl and nylon sutures, respectively. Sterile dressings were applied. Percutaneous Achilles tendon lengthening was done as a secondary procedure to correct the right ankle equinus, and a back slab splint was then applied to the ankle in neutral position.

### Rehabilitation

Physiotherapy was initiated 4 days post-operatively. At that point, the main findings were: she transferred from the bed and chairs to standing position a bit slowly; she ambulated independently using crutches and a non-weight bearing gait on the right foot; the right lower limb was ~ 3.5 cm shorter than the left lower limb; in supine position, the valgus angle at the knee was 6 degrees on the right and 4 degrees on the left side; passive right knee flexion was 10–90 degrees (10 degrees short of full knee extension) in sitting position, and the ankle was in a neutral position in a backslab; quadriceps activity was trace-to-absent in supine position, 3−/5 upon active knee extension in sitting position, and she was able to wiggle toes and dorsiflex the ankle within the backslab.

While observing orthopaedic precautions and rehabilitation protocol guidelines for corrective osteotomy and Achilles tendon lengthening, she underwent supervised physiotherapy twice per week for approximately 12 weeks. The treatment consisted of oedema reduction techniques; progressive stretching to the knee and ankle joints and calf and thigh muscles; muscle activation and strengthening exercises; gentle myofascial release to treat tight and painful soft tissue around the right knee, thigh, and calf; progressive gait training; and a home exercise programme to elevate the leg and exercise daily with assistance of the mother. Progressively aggressive ankle rehabilitation started approximately 4 weeks post-operatively, when the backslab was removed, and consisted of joint mobilization, stretching to the Achilles tendon, ankle muscle activation, and the use of pain and edema control modalities. The child was followed up at 6-month intervals.

## Results

### Pre-operative digital and physical planning workflow

The prototype was successfully printed at BITRI, and the model showed ripple-like features throughout, with hollow areas at the distal end of the femur and proximal aspect of the leg (fibula and tibia) (see Fig. 8). Virtual simulation of the surgeon’s proposed correction on the MD Studio showed good bone alignment (see video link in Section 4.2 below).

### Computer-aided fit validation and manufacturing

Similar to the virtual simulation of the surgeon’s proposed correction, the simulation of the designed implant on the MD Studio showed excellent patient implant fit (implant fit simulation-video 1_Slomo.mp4). The implant was successfully printed at CUT CRPM, and the mechanical tests conducted on the destructive test samples printed with the implant showed that the mechanical properties were within the minimum ASTM requirements for Class F tensile strength (x,y, and z direction) of 825 MPa and elongation of 8% (ASTM F3001 (2014) after thermal processing (Fig. 9A). The CT scan showed no major flaws at the scan resolution (Fig. 9B). The final manufactured and polished implant that was used for the surgical correction is shown below (Fig. 10).

### Surgery

The surgical procedures were completed in a total of 62 minutes. Immediately after fixation, limb alignment was visually checked, and mechanical axis was found acceptable. Plain X-ray imaging confirmed good implant fixation and positioning (Fig. 11).

### Rehabilitation

At the time the patient was discharged from supervised physiotherapy treatment, the right leg was ~ 3 cm shorter and both the genu valgus and equines deformities were corrected relatively well, with and without weight bearing, when compared to the contralateral side; while sitting, right ankle dorsiflexion was 0–15 degrees and comparable to the left side, and both planterflexion and knee flexibility were within normal limits; the low back, knee, ankle, and Achilles tendon were pain-free at rest, with activity, and during palpation using maximally tolerable pressure to each of these body parts; and the lower extremity functional score was 41/80 and associated with ability to squat relatively well (movement was unsupported and slightly slow), roll in bed, and stand for as long as desired; and she could ambulate for over 1 km without pain and without an assistive device, with slightly noticeable limping gait.

At each follow-up point, she declined a shoe insert that would address the residual leg-length discrepancy, arguing that her walking felt normal and comfortable. At the most recent follow-up (18 months post-surgery), she was pain-free, able to perform full squats without support (edited video squatting.mp4), walked almost normally without the aid of an assistive device (edited video walking.mp4), and scored 75/80 (93.8%) on the LEFS.

## Discussion

The aim of the project was to determine the feasibility of developing an effective, safe, and cost-effective patient-specific 3D-printed paediatric orthopaedic implant that could help improve post-surgical outcomes and the overall cost of care. The whole project (pre-surgical planning and design, manufacturing, surgery, and rehabilitation) is represented in the attached workflow link (Design process and project workflow.pdf). We have successfully demonstrated the feasibility of using Anatomage MD Studio software and 3D printing technology for pre-surgical planning, implant development, and corrective osteotomy in a paediatric patient with Ollier’s disease. The main findings were: (i) a reconstruction of the anatomical defect site was created from the patient’s CT scan, and implant fit and surgical technique were simulated using MD Studio; (ii) a surgical implant was successfully designed and fabricated in South Africa, iii) surgical reconstruction was successfully carried out on the patient, with proper implant fit and good bone and joint alignment, and iv) the child was successfully integrated back into school and the community, without pain and with good functional mobility.

Several medical and dental design software packages are commercially available. In their review article, Scherer et al. [14] reviewed Invivo (Anatomage; San Jose, Calif.), Simplant (Materialise NV; Leuven, Belgium), and NobelClinician (NobelBiocare; Yorba Linda, Calif.) for several features important in the process of computer-guided surgical planning and placement of implants; Invivo (MD studio incorporated) and Simplant Pro were comparable and highly rated. MD studio is a sophisticated, powerful software that allows the user to enhance the study of specific regions of the body and create anatomical models and simulations. This capability allowed us to simulate surgery and guide the surgical team with the proposed osteotomy cut angles and implant designs developed in the study. Comparison of the surgical outcome with the simulation video showed an accurate representation of the true outcome of the surgery.

Whereas the literature shows that Pre-surgical planning and 3D printing applications in medicine and dentistry are relatively at advanced stages in the developed world [14, 20–23], to the best of our knowledge, there are no published studies on the use of the MD Studio software in the pre-surgical planning and development of patient-specific medical devices in Africa. Although in Botswana and other developing countries, surgeons manually modify implants to desired shape, size, and fit, the capability of software like MD Studio, which allows for precise and relatively rapid production of custom implants, has long been known to help solve mismatch problems in orthopaedics [21, 22]. Our work, therefore, forms the first clinical use and application of this technology in Africa.

The printed prototype model had a ripple-like pattern/artefacts, which could be due to “conservative” scan slice settings selected for the child when CT images were being scanned. The model also had voids at the knee and abnormal surface on the lateral, distal end of the knee, believed to represent the abnormal cartilaginous tissue characteristic of the Ollier’s disease. These defects, however, did not affect the pre-surgical planning process and surgical outcome.

Results for tensile strength, elongation, and microcrack analysis demonstrate our ability to design and manufacture custom implants that meet ASTM requirements. The absence of physical manipulation of the implant by the surgeon to fit the unusual anatomical defect in this case allowed for a safe, structurally stable, and thus likely more durable implant because there would be no microcracks that are typically caused by the manipulation. The microcracks, for example, cause mechanical instability and corrosion, which overtime contribute to implant failure [8, 24] and subsequently create a need for earlier surgical revision. The greatly improved LEFS scores, resolution of back pain and falls, the video showing improved and advanced functionality, and re-integration into school and the community, all of which enhance self-confidence, corroborate the notion of a precisely fitting and effective implant. We think that our work may benefit Botswana and other countries, where the predominant use of off-the-shelf, standard-sized orthopaedic implants may result in implant-patient mismatch or implant failure.

Excluding the research team’s in-kind contribution, the total cost for the implant was approximately USD7,289.82 at the then local currency conversion rate. The cost covered the CT scan; printing of 2 prototypes (1 in Botswana, for use by the surgeon, and the other in South Africa for use at CUT for final implant design); printing of the final implant (including CT scanning, destructive testing, thermal analysis, shipping); and surgical screws. If all the design work had occurred in Botswana, the CUT prototype would not have been needed, thus reducing the final cost of the implant to about USD6,070.00. At the time of the surgery, the unit cost of generic off-the-shelf implants, which are mass-produced, was estimated at~USD1,300.00–2150.00, depending on the manufacturer.

Despite the apparently higher cost for our implant when compared to the generic implant, several arguments may be advanced in favour of our patient-specific implant. *First*, since paediatric implants and those for unique anatomical defects are not readily available locally, we think that the ability to customise implants, rather than use oversized or physically manipulated devices, is essential to providing quality healthcare. Challenges reported with the use of generic implants [3, 7, 8, 24] have the potential to result in future surgical revisions, which would greatly increase the cost of care. *Second*, cases with complex, anatomical defects are sometimes referred to South Africa, with the total cost of care often reaching tens of thousands of dollars. *Third*, the waiting time for some of the complex cases may be long due to limited resources, with or without referral to South Africa. Producing the implant in Botswana could greatly alleviate the problem of resource constraints and thus reduce the waiting time for some patients. *Finally*, we note that some of the various production works in the project were outsourced due to a lack of resources at the University of Botswana.

As previously suggested [21, 25–27], we believe that overtime, with increased demand for and production of this technology driven by adequate local/regional investment or funding, 3-D printing production costs could be greatly reduced [6]. This work underscores the importance of regional/international collaboration and partnerships between low-resourced and better-resourced institutions and countries, but in the long-term the aim should be to build and improve local capacity in manufacturing.

## Conclusion

Patient-specific treatment approaches incorporating 3D-printed implants are feasible, safe, and ultimately cost-effective in Botswana for carefully selected cases, where the conventional methods are not a viable or an appropriate option. Our novel work is a proof-of-concept and framework that can be used to encourage surgeons locally and regionally to treat complex or externally referred cases, with potential for decreasing national healthcare costs.
